# Transient abRNA and steady-state mRNA levels set the thresholds for sense PTGS initiation and amplification

**DOI:** 10.1093/plcell/koag245

**Published:** 2026-08-14

**Authors:** Hongwei Jing

**Affiliations:** Assistant Features Editor, The Plant Cell, American Society of Plant Biologists; Department of Horticultural Science, Kilgore Hall, 2721 Founders Dr, North Carolina State University, Raleigh, NC 27695, United States

Post-transcriptional gene silencing (PTGS) is a conserved RNA-based defense mechanism that protects plants against viruses and other invasive nucleic acids (Tan et al. 2020). During PTGS, double-stranded RNAs (dsRNAs) are processed by DICER LIKE 4 (DCL4) and DCL2 into 21- and 22-nucleotide small interfering RNAs (siRNAs), respectively, which are incorporated into ARGONAUTE (AGO)-containing RNA-induced silencing complexes (RISCs) that recognize and degrade homologous transcripts. In addition to local silencing, PTGS also generates mobile silencing signals that spread systemically throughout the plant (De Meo et al. 2025).

Sense post-transcriptional gene silencing (S-PTGS) is a gene-silencing process initiated by aberrant transcripts derived from highly expressed sense transgenes and requires RNA-DEPENDENT RNA POLYMERASE 6 (RDR6) for dsRNA synthesis, and DCL2 for secondary siRNA amplification (Vaucheret and Voinnet 2024). Because transgenes driven by the strong Cauliflower mosaic virus 35S promoter (Pro35S) are particularly prone to being targeted by this pathway, they have become a powerful genetic platform for dissecting RNA silencing mechanisms. Yet, despite their utility, how S-PTGS is initiated and how silencing signals are amplified to enable systemic transcriptional repression remain key unresolved questions in the field.

In a recent study, Martin Lacroix, Nicolas Butel, and colleagues (Lacroix et al. 2026) demonstrated that S-PTGS is governed by distinct thresholds: the initiation, involving transiently produced aberrant RNA (abRNA), and their amplification, involving high target mRNA levels (Fig. 1). To dissect the genetic pathways underlying systemic PTGS, the authors used a combination of transgenic and mutant lines. These included the *Pro35S:GUS* line *L1*, which undergoes S-PTGS in essentially all plants across generations, and the *jmj14* mutant, which lacks the Jumonji-C (JmjC) domain-containing protein 14 (JMJ14), a histone H3 lysine 4 di- and trimethyl (H3K4me2/3) demethylase involved in gene silencing. The authors also used the *JAP3* transgenic line, which expresses a hairpin RNA targeting a portion of the *PHYTOENE DESATURASE* (*PDS*) gene. Because *PDS* silencing produces a characteristic photobleaching phenotype, *JAP3* provides a convenient visual marker for monitoring PTGS.

**Figure 1:**
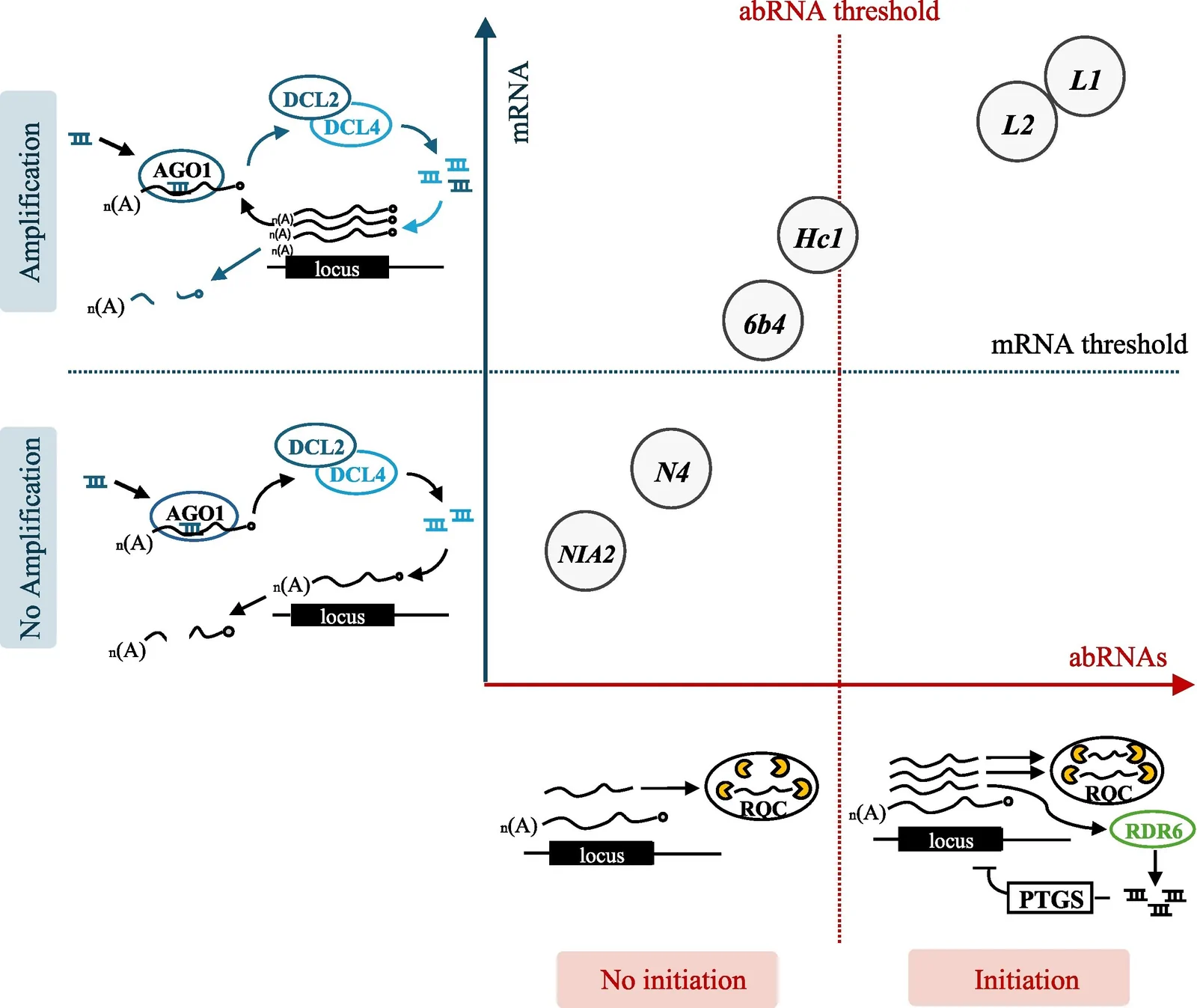
S-PTGS initiation and systemic amplification are governed by 2 distinct quantitative RNA thresholds. Initiation occurs when the quantity of aberrant RNAs (abRNAs) produced by a locus exceeds the initiation threshold, saturating the RNA quality control (RQC) machinery. This step is influenced by transcription rate, genomic context, and DNA arrangement, and likely occurs in only a small number of cells. The resulting primary siRNAs target homologous mRNAs. Amplification depends on a second, independent amplification threshold determined by the level of stable target mRNA. If target mRNA levels are low (below the threshold), the target mRNA are degraded and no secondary siRNAs are produced, DCL4 outcompetes DCL2, preventing amplification even if primary siRNAs move to adjacent cells. By contrast, if target mRNA levels are high (above the threshold), a fraction escape degradation and are converted into secondary siRNAs by RDR6, initiating a cycle of amplification that spreads systemically via mobile silencing signals. Adapted from (Lacroix et al. 2026), Fig. 6.

Previous studies showed that *jmj14* partially suppresses S-PTGS in the *L1* background (Le Masson et al. 2012). To further understand how JMJ14 promotes systemic S-PTGS, the authors sought to identify genetic factors that act in systemic PTGS. They first introduced the *JAP3* locus into *L1 jmj14* plants and performed an EMS (ethyl methane sulfonate) suppressor screen in search of mutations that restored GUS silencing and the associated photobleaching phenotype. This screen identified mutations in *AGO4* and *MORPHEUS′ MOLECULE 1* (*MOM1*), revealing these factors as negative regulators of S-PTGS in the *L1 JAP3 jmj14* background. Moreover, these mutations also suppressed the ectopic DNA methylation associated with JMJ14 deficiency. Together, these findings revealed a correlation between the capacity of the *Pro35S:GUS* transgene to undergo systemic S-PTGS and its transcriptional activity, which is influenced by DNA methylation at its promoter.

Further analysis of a large collection of independent *Pro35S:GUS* transgenic lines in a wild-type or PTGS-deficient mutant (*rdr3*) background revealed that most lines undergo systemic S-PTGS, as indicated by repression of GUS expression in systemic tissues. Only a small subset failed to initiate silencing, while an even smaller number were unable to amplify the silencing response once initiated. These observations suggest that the thresholds governing S-PTGS initiation and amplification are distinct.

Moreover, the *Pro35S:GUS 6b4* line, which normally fails to initiate S-PTGS but retains sufficient GUS target mRNA to support silencing amplification, provided a system to test the initiation threshold. In this line, localized and transient induction of GUS expression by *β*-estradiol was sufficient to initiate S-PTGS, which subsequently spread systemically. Thus, a transient increase in aberrant RNA above the initiation threshold can trigger systemic S-PTGS, even in a transgenic line that normally remains below this threshold.

The authors also tested whether the DCL4/DCL2 balance influences PTGS of endogenous genes. They grafted *dcl4* scions carrying wild-type *NITRATE REDUCTASE* (*NIA*) onto transgenic rootstocks in which *NIA* was silenced. The rootstock-derived silencing signal induced systemic PTGS of *NIA* in the scion, with silencing enhanced further in the *dcl4* background. This result supports a model in which loss of DCL4 favors DCL2-dependent production of secondary siRNAs, thereby promoting amplification and systemic spread of PTGS. Taken together, these findings reveal that distinct RNA thresholds govern the initiation and amplification of S-PTGS, with the latter also depending on the balance between DCL2 and DCL4 (Fig. 1).

Despite these important advances, several fundamental questions remain. What molecular features distinguish the S-PTGS-initiating aberrant RNAs from the abundant transcripts that normally escape RDR6-mediated S-PTGS initiation? How are the initiation and amplification thresholds quantitatively established and coordinated with chromatin state, transgene expression, and RNA metabolism? Furthermore, the mechanisms by which transient increases in aberrant RNA production are converted into robust systemic silencing signals remain unclear. Addressing these questions will provide a more comprehensive understanding of how plants balance transgene expression with RNA surveillance and may facilitate the rational manipulation of PTGS to improve transgene stability, antiviral immunity, and RNA-based crop biotechnology.

## Recent related articles in *The Plant Cell*

- Kramer and coauthors (Kramer et al. 2025) identified a specific aberrant RNA that triggers transgene silencing, showing ribosome stalling at a histidine motif initiates No-Go RNA Decay and produces a cleavage product of the RUBY mRNA that precedes RNAi.
- Qi and colleagues (Qi et al. 2026) identified the spliceosome component PRP8 as a key regulator of transcriptional gene silencing that functions independently of DNA methylation by maintaining higher-order chromatin organization.
- Li and colleagues (Li et al. 2024) identified ALTERED MERISTEM PROGRAM1 (AMP1) and its paralog LAMP1 as negative regulators of inverted repeat (IR)-derived siRNA biogenesis in Arabidopsis, acting by suppressing ARGONAUTE1 (AGO1) protein levels to inhibit Pol II-dependent IR transcription.
- Bélanger and coauthors (Bélanger et al. 2023) discovered that plastid-expressed dsRNA transgenes can escape to the cytoplasm and trigger post-transcriptional gene silencing of nuclear-encoded genes via the biogenesis of 21-nucleotide phased small interfering RNAs (phasiRNAs).

## Data Availability

No new data were generated or analyzed in support of this research.
